# Comparative whole genome sequence analysis of wild-type and cidofovir-resistant monkeypoxvirus

**DOI:** 10.1186/1743-422X-7-110

**Published:** 2010-05-28

**Authors:** Jason Farlow, Mohamed Ait Ichou, John Huggins, Sofi Ibrahim

**Affiliations:** 1 Virology Division, U.S. Army Medical Research Institute of Infectious Diseases, Fort Detrick, Frederick, MD 21702-5011, USA

## Abstract

We performed whole genome sequencing of a cidofovir {[(S)-1-(3-hydroxy-2-phosphonylmethoxy-propyl) cytosine] [HPMPC]}-resistant (CDV-R) strain of Monkeypoxvirus (MPV). Whole-genome comparison with the wild-type (WT) strain revealed 55 single-nucleotide polymorphisms (SNPs) and one tandem-repeat contraction. Over one-third of all identified SNPs were located within genes comprising the poxvirus replication complex, including the DNA polymerase, RNA polymerase, mRNA capping methyltransferase, DNA processivity factor, and poly-A polymerase. Four polymorphic sites were found within the DNA polymerase gene. DNA polymerase mutations observed at positions 314 and 684 in MPV were consistent with CDV-R loci previously identified in Vaccinia virus (VACV). These data suggest the mechanism of CDV resistance may be highly conserved across *Orthopoxvirus *(OPV) species. SNPs were also identified within virulence genes such as the A-type inclusion protein, serine protease inhibitor-like protein SPI-3, Schlafen ATPase and thymidylate kinase, among others. Aberrant chain extension induced by CDV may lead to diverse alterations in gene expression and viral replication that may result in both adaptive and attenuating mutations. Defining the potential contribution of substitutions in the replication complex and RNA processing machinery reported here may yield further insight into CDV resistance and may augment current therapeutic development strategies.

## Background

Poxviruses are large, enveloped, pleomorphic dsDNA viruses that infect a diverse array of mammals, reptiles, and insects [1]. The causative agent of Smallpox, Variola virus (VARV) is a member of the OPV genus. Smallpox was declared eradicated in 1980, however, natural or illicit re-emergence poses a risk for a growing non-vaccinated population [2]. MPV is a re-emerging pathogen within the OPV genus that causes sporadic outbreaks in monkeys and humans in West and Central Africa and, recently, in North America [3]. MPV can cause human disease clinically similar to Smallpox but with lower morbidity and mortality rates [4]. Although terrestrial and arboreal rodents and mammals are thought to play a role in MPV transmission, human to human transmission is known to occur [5].

Poxviruses possess large, complex genomes that encode their own viral replication machinery in addition to a plethora of immunomodulating proteins [1]. The major components of the poxviral replication complex include the poxvirus DNA polymerase (DNApol, E9L), transcription factor heterodimer (vETF), DNA-dependent RNA polymerase, RNA polymerase accessory protein (RAP94), viral poly-A polymerase (VP55/VP39), capping methyltransferase (D1/D10), and the DNA polymerase processivity factor (A20) [1,6]. Chemotherapeutic strategies for poxvirus infection have largely targeted viral DNA synthesis in order to disrupt the virus replication cycle [7,8].

A number of nucleoside/nucleotide analogs are available that inhibit OPVs [7]. The acyclic nucleoside phosphonate analogue (*S*)-1-[3-hydroxy-2-phosphonyl-methoxypropyl)] cytosine ((*S*)-HPMPC) or cidofovir (CDV) has been shown to inhibit *in vitro *viral replication of most known DNA viruses including poxviruses [9-11]. Recent studies suggest a mechanism whereby CDV may allosterically reposition the 3' nucleophile of terminal and short +strand synthesis products leading to aberrant chain extension [12,13]. Using the VACV DNApol E9L, previous studies indicate CDV incorporation slows chain elongation and inhibits DNA synthesis [12]. In addition, CDV has been shown to inhibit 3' to 5' exonuclease activity of E9L when incorporated in the penultimate position relative to the primer terminus [12]. By altering chain extension CDV affects DNA synthesis, a key regulator of poxvirus gene expression. Thus, alterations in gene expression and replication are likely to occur during CDV exposure, and, could result in mutations affecting conserved determinants of the virus life cycle.

Cidofovir activity appears to be conserved in dsDNA viruses providing a common strategy for inhibiting viral replication in important human diseases caused by these virus families [14,8,15]. Substitutions in the DNApol exonuclease (A314T) and polymerase (A684V) domains of the VACV DNA polymerase have previously been mapped and shown to confer CDV resistance [16,17]. CDV resistant strains in other members of the OPV genus, including MPV, Camelpoxvirus (CMPV), and Cowpoxvirus (CWPV) have already been reported [15]. DNApol mutations conferring resistance to CDV may be conserved among non-VACV OPV species although, presently, such sequence analyses have not been performed. Indeed, a portion of resistance attributes are likely to be conserved across dsDNA viruses. A number of additional features of CDV-resistance remain uncharacterized. CDV resistant strains frequently display an attenuated phenotype [18,15] through yet uncharacterized natural genetic alterations. In addition, it has been suggested that, in some cases, resistance to CDV requires mutations outside the DNA polymerase. One previous study identified a CDV-R VACV which exhibited a single non-essential substitution in the DNApol that upon reconstruction did not confer CDV resistance [18]. To date, such loci elsewhere in the genome remain unknown. Whole-genome sequence data could provide valuable insight into breadth of mutations induced by CDV exposure and yield insight into further requisites for attenuation and resistance.

We report here the first whole genome sequence of a CDV-R poxvirus. Our data revealed a plethora of substitutions within the CDV-R MPV genome, one-third of which were distributed throughout the viral replication machinery. Substitutions identified in the MPV DNA polymerase are consistent with those previously observed in VACV suggesting CDV-resistance determinants may be conserved in the OPV genus. The numerous substitutions observed throughout the replication and RNA processing machinery suggest multiple accrued mutations may alter the timing and regulation of the virus life cycle under CDV exposure. Novel loci reported here may inform future studies aimed at mechanistic interaction of CDV with the replication complex.

## Results and Discussion

**Figure 1:**
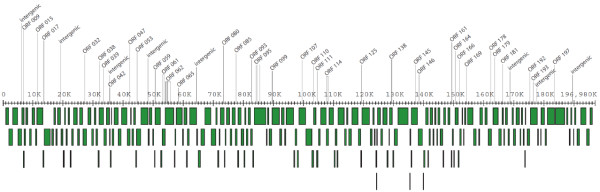
**Physical location of MPV CDV-R substitutions and indels in the MPV Zaire 1979-005 genome**. Gene spacing is based on NCBI graphics output http://www.ncbi.nlm.nih.gov/nuccore/68449077?report=graph&log$=seqview. Open reading frames (ORFs) corresponding to sites listed in Table 1 are noted above horizontal axis.

**Figure 2:**
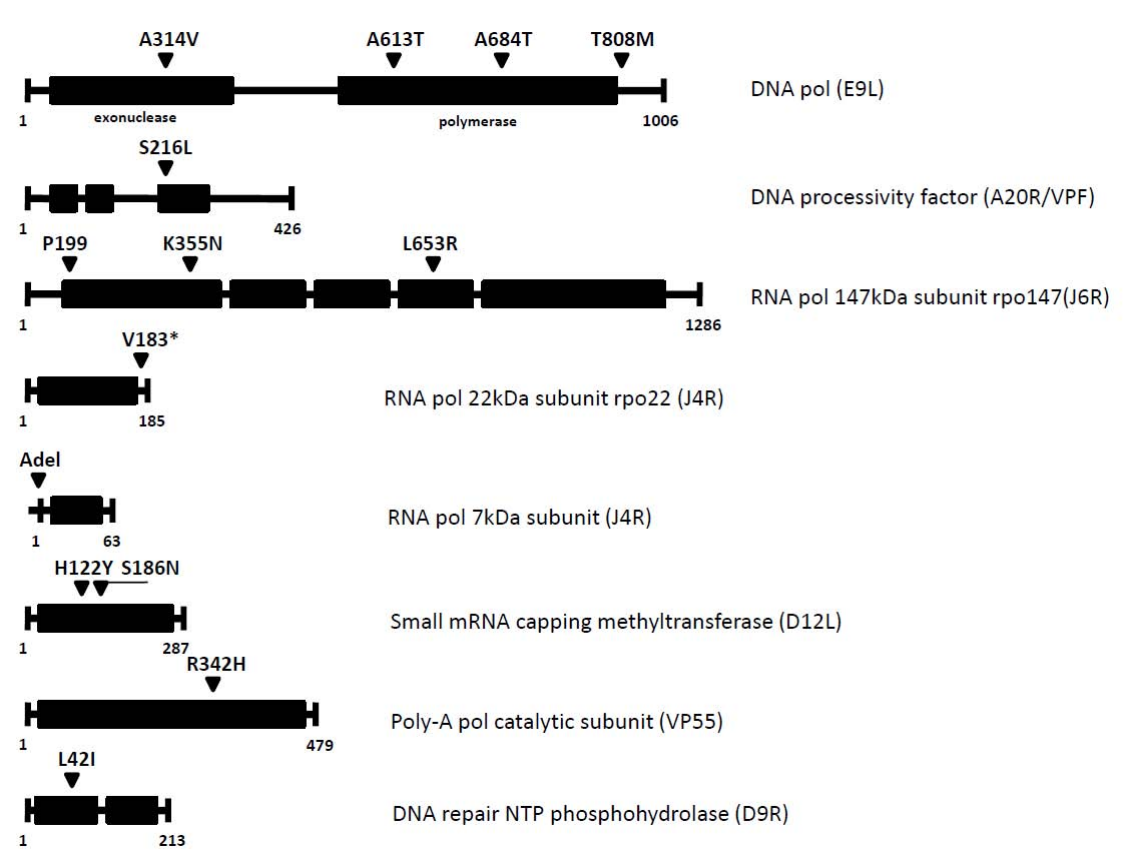
**Viral replication-associated amino acid substitutions from Table 1**.

Whole genome comparison of CDV-R and WT strains of Monkeypox revealed 55 single nucleotide polymorphisms (SNPs) including four insertions, six deletions, and 44 nucleic acid substitutions (Table 1, Figure 1, 2). A total of 10 intergenic and 45 intragenic SNPs, were observed that include 17 synonomous, 26 nonsynonomous substitutions and one tandem repeat contraction (Table 1). Over a third of all observed SNPs occurred within genes involved in virus replication and DNA metabolism. The physical distribution of all observed SNPs and indels (insertions/deletions) are illustrated in Figure 1.

**Table 1:** Genome-wide SNP/indel attributes of CDV-R MPV.

Location^*a*^	Mutation	Amino Acid	Z79-ORF^*b*^	COP-ORF^*c*^	Gene	GenBank#
6166	T insertion	NA	IG^*d*^	NA	NA	NA
6863	G to A	P9S	9	unknown	ankyrin-like	AAY97204
11360	T insertion	NA	15	unknown	ankyrin/host Range	AAY97210
13685	C to T	A589T	17	C9L	ankyrin	AAY97212
19143	C to T	NA	IG^*d*^	NA	NA	NA
27141	C deletion	A168Q	32	K2L	serine protease inhibitor-like protein SPI-3	AAY97227
32192	T deletion	H385L	38	F3L	Kelch-like	AAY97233
33518	C to T	silent	39	I4L/F4L	ribonucleoside-diphosphate reductase	AAY97234
35560	T deletion	NA	IG^*d*^	NA	NA	NA
35593	G to A	T68M	42	F7L	unknown	AAY97237
40212	C to A	silent	47	F12L	IEV associated	AAY97242
44808	C to T	R342H	53	E1L	poly-A polymerase catalytic subunit VP55	AAY97248
48134	A to T	NA	IG^*d*^	NA	NA	NA
50728	T to A	silent	59	E6R	unknown	AAY97254
53128	T to C	silent	61	E8R	assoc.s with IV/IMV and cores; F10L kinase substrate	AAY97256
53738	G to A	V256I	61	E8R	assoc.s with IV/IMV and cores; F10L kinase substrate	AAY97256
54066	T to C	silent	62	E9L	DNA polymerase	AAY97257
54400	G to A	T808M	62	E9L	DNA polymerase	AAY97257
54773	C to T	A684T	62	E9L	DNA polymerase	AAY97257
54986	C to T	A613T	62	E9L	DNA polymerase	AAY97257
55882	G to A	A314V	62	E9L	DNA polymerase	AAY97257
58655	T to C	silent	65	01L	unknown	AAY97260
64425	A insertion	NA	IG^*d*^	NA	NA	NA
73563	A deletion	NA	IG^*d*^	NA	NA	NA
77811	G to A	M207I	85	L1R	myristylprotein	AAY97280
82937	C to T	silent	93	J4R	DNA-dependent RNA polymerase subunit rpo22	AAY97288
84110	G to A	silent	95	J6R	DNA-dependent RNA polymerase subunit rpo147	AAY97290
84578	A to C	K355N	95	J6R	DNA-dependent RNA polymerase subunit rpo147	AAY97290
85471	T to G	L653R	95	J6R	DNA-dependent RNA polymerase subunit rpo147	AAY97290
89604	G to A	silent	99	H4L	RNA polymerase-assoc. transcription factor RAP94	AAY97294
89691	C to T	M715I	99	H4L	RNA polymerase-assoc. transcription factor RAP94	AAY97294
99891	T to C	silent	107	D5R	NTPase, DNA replication	AAY97302
103281	C to T	A289T	110	D8L	carbonic anhydrase/Virion	AAY97305
104948	C to A	L42I	111	D9R	nudix-hydrolase/RNA decapping	AAY97307
107809	C to T	H122Y	114	D12L	small capping enzyme, methyltransferase	AAY97309
108002	G to A	S186N	114	D12L	small capping enzyme, methyltransferase	AAY97309
119244	G to A	silent	125	A9L	membrane protein	AAY97320
129030	C to T	S216L	138	A20R	DNA processivity factor	AAY97333
129340	G to A	silent	138	A20R	DNA processivity factor	AAY97333
137047	A to G	L324S	145	A25L	A type inclusion protein (CPXV)	AAY97340
138486	ATCATC deletion	DD-del^*e*^	146	A26L	P4c: CWPVA27L, A-type inclusion protein	AAY97341
149213	G to A	silent	161	A42R	profilin homolog	AAY97356
150527	C to T	A284T	164	A44L	bifunctional hydroxysteroid dehydrogenase	AAY97356
151960	T to C	silent	166	A46R	IL-1 signaling inhibitor	AAY97361
154086	G deletion	frameshift	169	A48R	thymidylate kinase	AAY97364
162118	C to T	H268Y	178	B2R/B3R	Schlafen ATPase	AAY97370
163857	C to T	A271V	179	B4R	ankyrin	AAY97371
166078	A to T	Q9H	181	B6R	ankyrin	AAY97373
168859	T to A	NA	IG^*d*^	NA	NA	NA
175168	C to T	silent	192	B18R	IFN-α/β-receptor orthologue	AAY97385
176348	T to C	silent	193	unknown	ankyrin	AAY97386
177838	T insertion	NA	IG^*d*^	NA	NA	NA
183499	T to C	silent	197	CWP_B22R	surface glycoprotein	AAY97391
189631	T to C	NA	IG^*d*^	NA	NA	NA
190055	A deletion	NA	IG^*d*^	NA	NA	NA

^*a *^indicates position of mutation relative to the MPV Zaire 1979-005 genome sequence (DQ011155.1). ^*b *^indicates open reading frame (ORF) designations within the Zaire-1979-005 genome. ^*c *^specifies open reading frame designations within the VACV Copenhagen genome (M35027.1). ^*d *^designates an intergenic non-coding locus. ^*e *^designates deletion of two aspartic acid residues (D) from the c-terminal poly D repeat of gene 164 (homologue of VACV A26l).

### DNA replication

**Figure 3:**
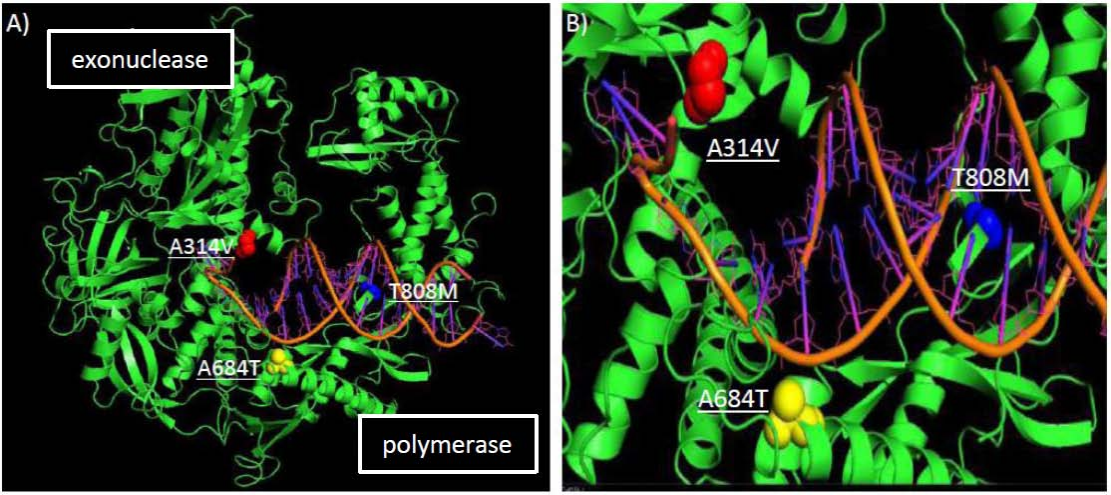
**MPV CDV-R mutations mapped onto the 3D structures of herpes simplex 1 DNA polymerase**. Mutations A314V (red), A684T (yellow), and T808M (blue) are illustrated in view of the entire protein (A) and DNA binding cleft (B).

Poxviruses exert exquisite control over the timing of gene expression to regulate genome replication and virion assembly [19]. Five early proteins are essential for poxvirus DNA replication, including the DNA polymerase (E9), DNA-independent nucleoside triphosphatase (NTPase, D5), uracil DNA glycosylase (D4), protein kinase B1, and DNA processivity factor (VPF/A20) [19,6]. In our study, substitutions were observed in the 3' to 5' exonuclease and 5' to 3' polymerase domains of the MPV DNA polymerase (Table 1, Figure 2, Figure 3A, B) consistent with previous studies in VACV [10,12,20]. A total of four non-synonomous substitutions and 1 synonymous substitution were observed in the MPV DNA polymerase gene (ORF 062) (Table 1). The CDV-R MPV DNApol encoded substitutions A314V and A684T at conserved positions respective to CDV-R VACV [16], although the substituted residues appear reversed (MPV = V314/T684, VACV = T314/V684). In both cases, A314 and A684 in MPV and VACV are replaced by slightly larger residues with differing polar characters (threonine = +4.9, valine = -2.0). Two novel substitutions A613T and T808M in the MPV CDV-R strain were located within and flanking the polymerase domain, respectively (Figure 2).

**Figure 4:**
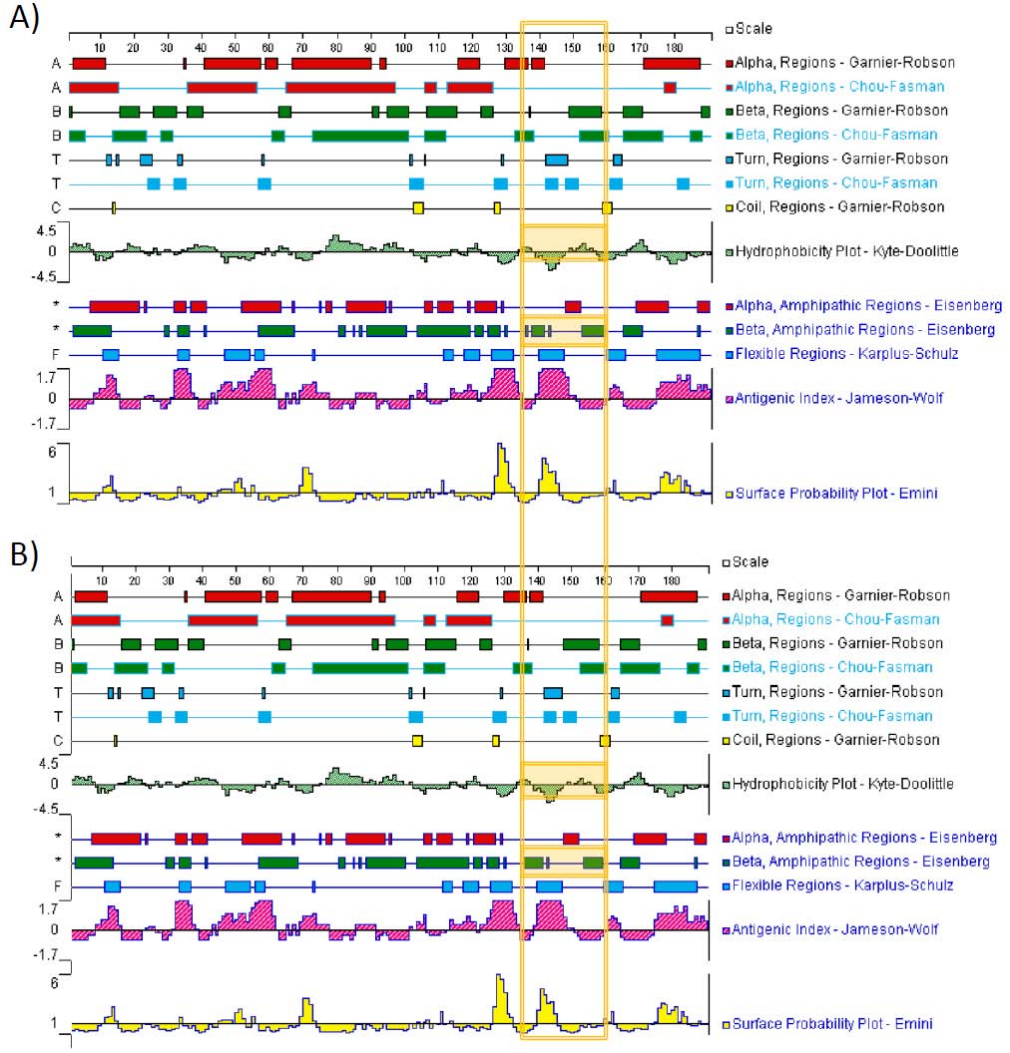
**Topological feature maps of CDV-R (A) and WT (B) MPV DNA pol 3'-5' exonuclease domain**. Plotted residues 1-190 correspond to 162-351 in the MPV DNA pol exonuclease domain. The A314V substitution (Table 1) corresponds to position 153 in the plot. For comparison, regions of difference in secondary structure and biochemical characteristics between CDV-R and WT are designated by shaded areas in the vertical orange box.

**Figure 5:**
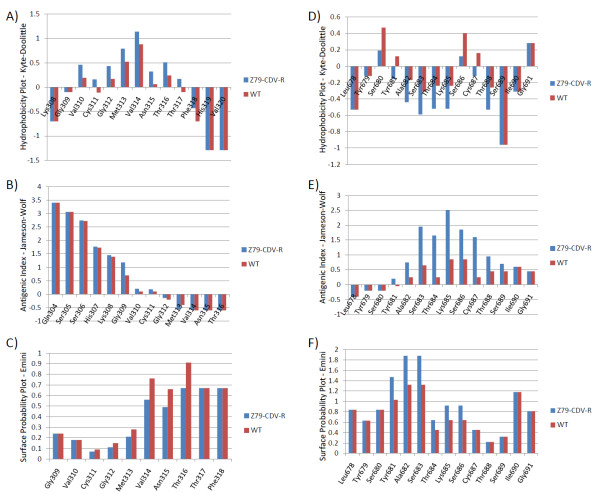
**Biochemical and surface prediction plots of MPV CDV-R and WT DNA pol substitutions**. Features of the A314V locus are presented in plots A-C, and A684T in plots D-F.

**Figure 6:**
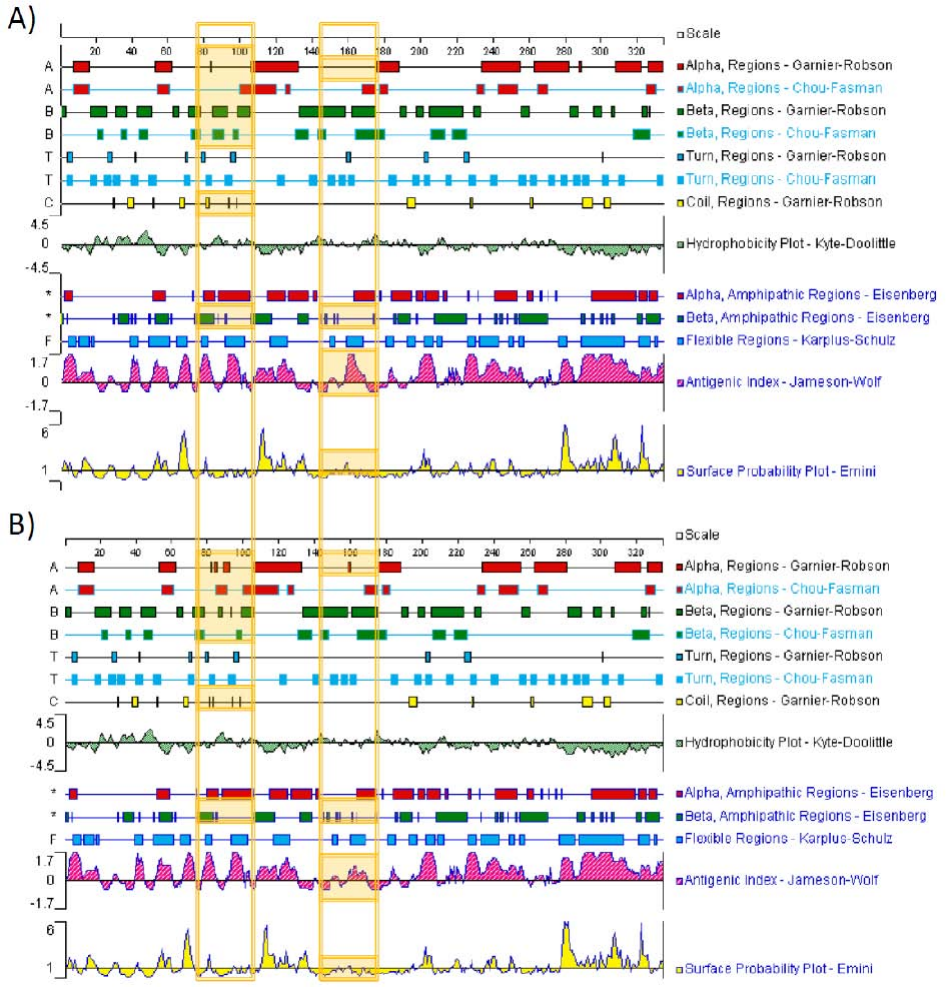
**Topological feature maps of CDV-R (A) and WT (B) MPV DNA pol domain**. type B DNA polymerase residues 525-806, T808M = T284,. Plotted residues 1-330 correspond to residues 525-806 in the MPV DNA pol catalytic domain. The A613T, A684T, and T808M substitutions (Table 1) correspond to positions 89, 160, and 284 in the plot. For comparison, regions of difference in secondary structure and biochemical characteristics between CDV-R and WT are designated by shaded areas in the vertical orange box.

We utilized predictive modeling software to extrapolate potential structural changes mediated by these substitutions in the MPV DNA polymerase protein. Predicted topological features of the CDV-R DNA polymerase A314V substitution in the exonuclease domain appears to increase the regional hydrophobicity, alter surface contour and decrease surface exposure (Figure 4A, B, Figure 5A, B, C, Table 2) at this locus. The A684T substitution in the polymerase domain appears to exhibit a decrease in the regional hydrophobicity (Figure 5D) and an increase in surface contour and exposure (Figure 5E, F), including a predicted shift from alpha helical to beta sheet topology (Figure 6A, B). Similar analysis suggests a slight increase in surface exposure at the A613T locus and a moderate loss of surface exposure at the T808M locus (Table 2). It has been hypothesized that the resistant mutation at the A314 locus in the exonuclease domain may facilitate excision of CDV during replication, while mutation at A684, located adjacent to the DNA-binding pocket (Figure 3A, B), may be involved in nucleotide selection and discrimination of CDV [20]. Solving the 3-D structure of a poxvirus DNApol may provide further clarity on the positional activity and functional attributes of these mutations.

**Table 2:** Biochemical and topological attributes of CDV-R MPV mutations

Protein	ORF^*a*^	Amino Acid	Domain	Δ Polarity^*b*^	Δ Hydropathy^*b*^	Surface Exposure^*d*^	Surface Contour^*e*^
DNA pol	E9L	A314V	exonuclease	0.1	2.4	decrease	increase
DNA pol	E9L	A613T	polymerase	6.8	2.5	increase	increase
DNA pol	E9L	A684T	polymerase	6.8	2.5	increase	increase
DNA pol	E9L	T808M	NA	6.4	2.6	decrease	decrease
RNA pol subunit rpo147	J6R	K355N	TFIIB docking	5.3	3.12	decrease	increase
RNA pol subunit rpo147	J6R	L653R	funnel	22.3	8.3	increase	increase
mRNA capping enzyme small subunit	D12L	H122Y	dimerization	4.2	1.9	increase	decrease
mRNA capping enzyme small subunit	D12L	S186N	dimerization	4.6	2.7	increase	decrease
poly-A pol catalytic subunit VP55	E1L	R342H	dimerization	9.7	0.7	decrease	decrease

^*a *^specifies ORFs relative to Copenhagen strain. ^*b *^changes in polarity and hydropathy due to amino acid substitutions were calculated using Kyle and Doolittle algorithm in Lazergene (DNAstar) software. ^*d *^surface exposure and ^*e *^contour were determined using the Emini method and Jameson-Wolf algorithm, respectively (DNAstar software).

### DNA processivity factor

Fully processive DNA polymerase activity is mediated by the heterodimeric A20/D4 DNA processivity factor [21]. A20 is essential for genome replication and may form a multi-enzyme replication complex with D4, D5, and H5 that is postulated to stabilize the DNA replication complex [22]. D5R is a nucleic acid independent nucleoside triphosphatase (NTPase) that is crucial for infection [23,24] and may play a role in priming DNA synthesis at the replication fork [25]. In our study, CDV-R MPV exhibited a substitution in A20 (S216L) that lies directly within the D5 NTPase/primase binding domain (Table 1, Figure 2) [22,26].

### Thymidylate kinase

**Figure 7:**
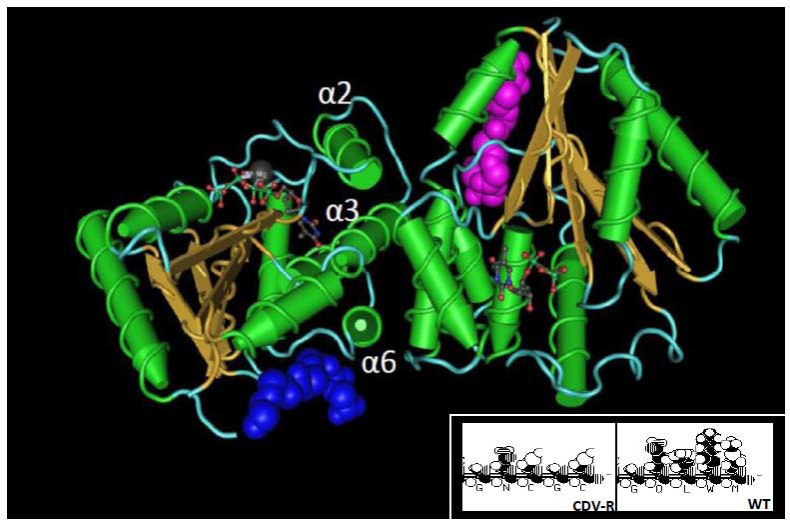
**MPV CDV-R c-terminal amino acid deletion mapped on 3-D structure of VACV thymidylate kinase (TMPK) homodimer**. The four residues corresponding to the c-terminal frameshift mutation in MPV CDV-R are labeled in blue and pink. Illustrations were prepared using Cn3D. Inset includes space-filling model of the four c-terminal residues of WT and CDV-R MPV TMPK (prepared using Lasergene software).

The poxvirus thymidylate kinase (TMPK) encodes a 48 kDa serine threonine protein kinase (A48R) [27] that regulates deoxyribonucleotide triphosphate pools in conjunction with the viral thymidine kinase. Similar to cellular TMPK, A48R functions as a homodimer where dimerization is mediated by proper orientation of the α2, α3, α6 helices [28]. The quaternary structure of A48R is distinct in orientation from that of the host conferring broader substrate specificity [28]. We observed a SNP deletion at residue 600 in the CDV-R MPV gene that results in a frameshift mutation at amino acid Q201 and replacement of the c-terminus residues "QLWM" with residues "NCGC" (Table 1, Figure 7 and inset). The frameshift results in a more pronounced turn region conferred by the proximal P198 predicted by chou-Fasman and Gernier-Robson algorithms (data not shown). This alteration may affect the dimerization interface of the homodimer given that the c-terminal residues support the α6 helix which mediates dimerization (Figure 7)[28]. It is interesting to speculate whether such a change in secondary structure could affect protein function during CDV exposure, such as discriminatory selection between CDV diphosphate and cellular dCTP pools.

### RNA polymerase machinery

The primary components of the poxviral RNA machinery consist of the poxvirus DNA-dependent RNA polymerase (rpo147), the viral early transcription factor vETF (D1/D12) heterodimer, eight RNA polymerase subunits, RAP94, VP55/VP39 subunits of the viral poly (A) polymerase, the capping methyltransferase (D1/D12), and the D9 subunit of the mRNA decapping enzyme (Table 1, Figure 2) [1]. Proteins RAP94, NPHI (D11), and D1/D12 constitute early termination factors [19]. Poxvirus RNA pol contains eight common subunits including rpo147, rpo132, rpo35, rpo30, rpo22, rpo19, rpo18, rpo7 [1]. The dual functional ninth subunit, RAP94, is absent in intermediate and late replication complexes [29] and is thought to function as an early transcription factor docking platform [30,31]. Vaccinia Early Transcription Factor (VETF), comprising D6R and A7L, binds to early promoters, recruits RAP94-containing RNA pol, and nucleates a stable pre-initiation complex at the early promoter [31]. Viral mRNA capping and addition of poly(A) tails are generated by the heterodomeric proteins D1/D12 and VP55/VP39, respectively [32-34]. In addition, cellular RNA pol II and TATA-binding proteins (TBPs) are recruited to poxvirus replication complexes, possibly to early and late viral promoters that show similarity to cellular RNA pol II TATA-box promoters [35,36]. Roles for such host proteins in the viral life cycle remain unknown. Several poxviral RNA polymerase subunits share limited sequence similarity with cellular RNA pol II subunits [36]. Previous studies indicate the largest subunit of the poxvirus RNApol (rpo147) exhibits the greatest homology to cellular RNApol II [37,38] while vaccinia VETF (D1-D12) and RAP94 show sequence similarity to cellular TBP-TFIID and RAP30-TFIIF, respectively [39]. In this study, we observed amino acid substitutions in MPV RNA pol II subunits including rpo147 (K355N, L653R), RAP94 (M715I), VP55 (R342H), D12 (H122Y, S186N), and D9 (L42I) (Table 1, Figure 2).

### RNA polymerase rpo147

**Figure 8:**
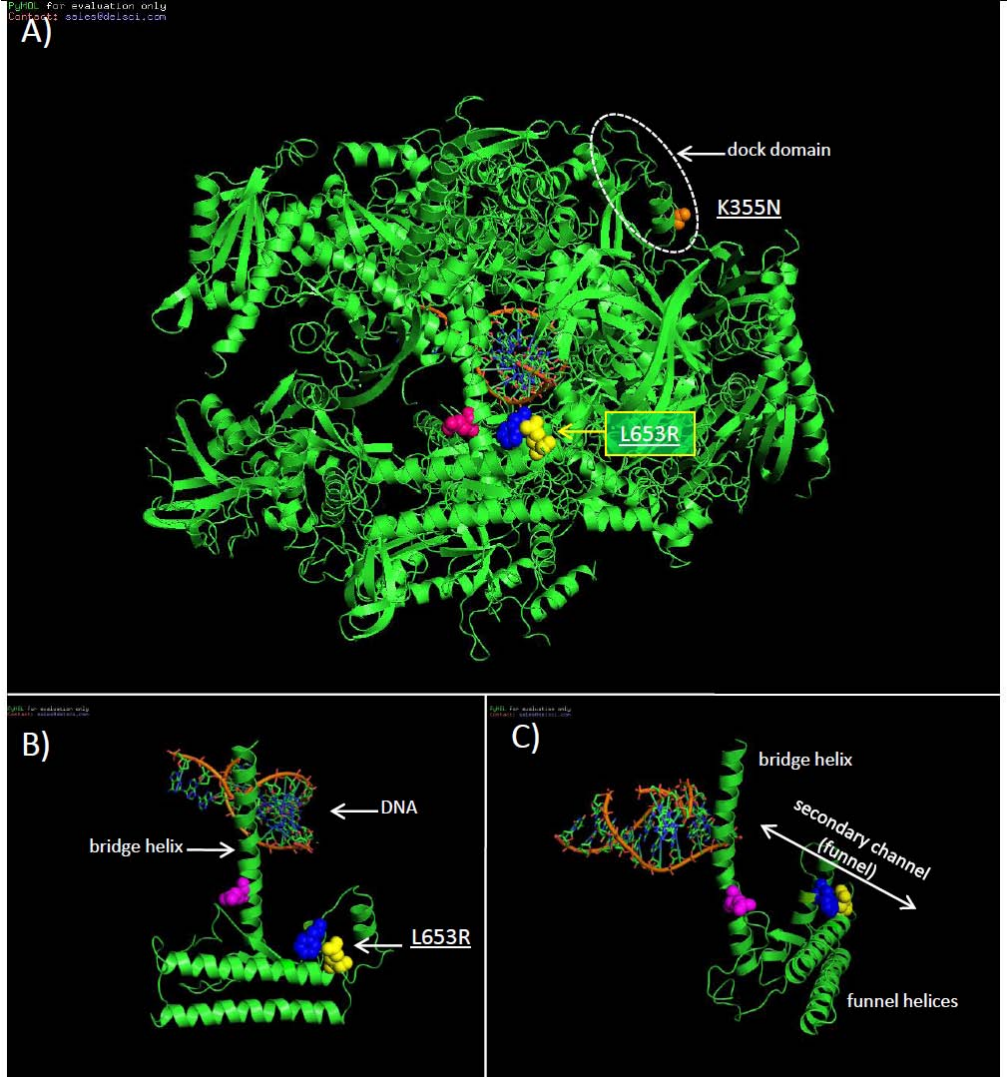
**MPV CDV-R substitutions mapped onto the 3-D structure of *S. cerevisiae *RNA pol II (GenBank # **CAA65619.1**)**. The MPV CDV-R L653R residue mapped to the yeast RNA pol II funnel domain is designated in yellow (A-C). The MPV CDV-R K355N residue mapped to the docking domain of yeast RNA pol II is designated in orange. Yeast residues Leu737 (blue) and Phe755 (magenta) are associated with α-amanitin resistance [46]. Illustrations were prepared using PyMol.

**Figure 9:**
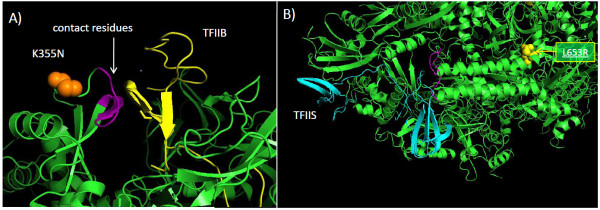
**MPV CDV-R RNA pol substitutions mapped onto the 3-D structure of *S. cerevisiae *RNA pol II**. CDV_R substitution K355N (orange) and L653R (yellow) mapped to the 3-D structure of A) binding sites of TFIIB (purple) on yeast RNA pol II and B) TFIIS (teal) [70], respectively. Illustrations were prepared using PyMol.

The L653R substitution in the poxvirus rpo147 subunit lies directly in a homologous region of domain 4 in the yeast RNA polymerase II (RNA pol II) Rpb1 subunit (yeast E734R) that comprises the funnel (secondary channel) domain (Figure 8A, B, C) [40]. The domain lies at the juncture of the catalytic domain and the outside medium and is thought to mediate NTP entry and selection and support exonuclease proofreading [40]. The funnel domain may mediate binding RNA cleavage stimulatory factor TFIIS (Figure 9B) [41], which stimulates RNA pol II nuclease activity following transcriptional arrest [42] and recruits RNA pol II and TFIIB to the promoter [43]. In addition, this domain is also the binding site for antimicrobial RNA pol inhibitors including α-amanitin and targetoxin [44-46]. The MPV CDV-R L653R substitution lies adjacent to residues previously shown to mediate cellular RNA pol II inhibitor α-amanitin resistance (Figure 8B and 8C) [45]. Protein structure prediction indicates the L653R mutation may decrease regional hydrophobicity, and increases motif surface exposure (Table 2). The extent of homology of poxviral rpo147 and rpo30 with cellular RNA pol II Rpb 1 and TFIIS [38,47] suggest general features of their interaction may be conserved.

The MPV CDV-R K355N substitution (yeast G422) lies directly within the docking domain near the RNA exit groove of RNA pol II (Figure 8A and 9A)[48]. The RNA pol II docking domain binds TFIIB through contact residues 407-RDSGDRIDLRYSK-419 located within a larger conserved 67 amino acid motif [48]. The MPV CDV-R K355N mutation lies within the docking domain (in purple) immediately adjacent to the contact residue motif (Figure 9A). A significant change in predicted secondary structure is imparted by the K355N substitution including a pronounced increase in the surface contour (Table 2). The effect of CDV on the viral and cellular RNA polymerase machinery has not been evaluated. It is possible that viral RNA pol may be subject to either direct or indirect effects of CDV via dCTP selection in the presence of CDV or transcriptional arrest due to disrupted mRNA transcripts. In any case, alteration of the functional activity of either the funnel or docking domain could significantly alter pre-initiation complex formation and affect transcriptional regulation and promoter recruitment.

### Capping methyltransferase

The poxvirus mRNA capping machinery, encoded by the D1R and D12L genes in VACV, catalyzes viral mRNA capping and regulates gene transcription [49,50]. The D1/D12 heterodimer mediates 5' methylation of viral transcripts [32], promotes early gene transcription termination [51], and regulates initiation of intermediate gene expression [52]. Methyltransferase (MT) catalysis is mediated by the C-terminal active domain of D1R. Triphosphatase and quanylyltransferase activity are located within the N-terminal domain [53]. Following heterodimerization, the stimulatory D12 subunit confers full D1R MT activity by stimulating MT catalysis up to 50 fold [54,55].

**Figure 10:**
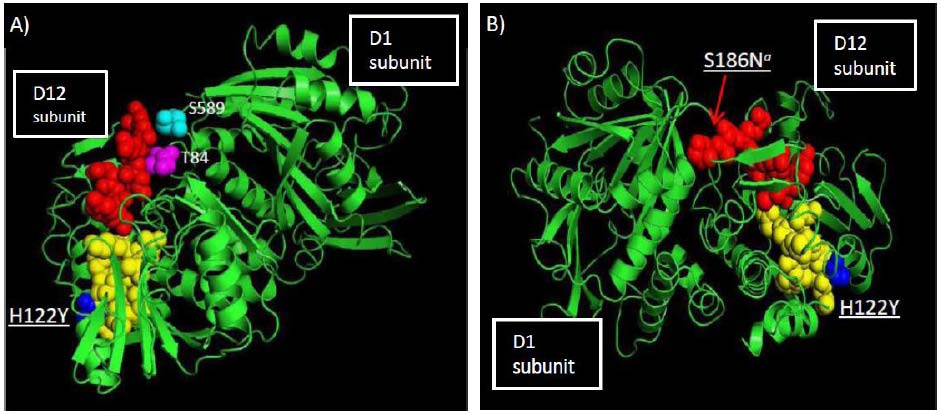
**MPV CDV-R mutations mapped onto the 3D structures of the poxvirus D1/D12 mRNA capping enzyme**. S186 which lies within the conserved motif 183-KCVSDSWLKDS (F, red residues) plays a major role in proper tertiary folding [54]. Yellow residues (E and F) designate the D12 hetero-dimerization motif [54]. D1 residues T84 (magenta) and S589 (teal) specify residues in closest proximity to the D12 S186N substitution (E). Illustrations were prepared using PyMol.

We observed two substitutions (H122Y and S186N) in the MPV CDV-R strain D12 orthologue (ORF114) (Table 1, Figure 2). Both substitutions lie within structural motifs that mediate allosteric interactions important for D1-D12 heterodimerization and MT activity (Figure 10A and 10B, in red and yellow) [53,56]. The basic H122 residue flanks two neutral residues, 120N and 121N, that affect important polar interactions between D1 and D12 (Figure 10A and 10B, in blue)[53,56]. CDV-R residue Y122 lies directly within an 11-aa motif (119-130) in the central domain region that plays a direct role in heterodimerization (yellow residues shown in Figure 10A and 10B) [53]. In addition, this short motif forms inter-subunit contacts with the D1R N-terminal α-Z helix and is proposed to allosterically stabilize substrate binding by D1R [53]. Predicted changes in secondary structure due to the H122Y substitution indicate a beta strand reduction (data not shown) and decreased surface contour and exposure (Table 2). Residue S186 lies with the conserved motif 183-KCVSDSWLKDS (red residues Figure 6F) that was previously noted as a highly structured motif which integrates several local and distal interactions which may play a major role in proper tertiary folding [53]. This position also flanks motif 189-WLKDS that may constitute a portion of the D1 subunit docking site [53]. S186 is in closest proximity to D1 residues S589 (teal) and T84 (magenta) (Figure 10A) and lies near the D1-D12 interface (Figure 10B).

D12 structurally stabilizes D1 through allosteric interactions that mediate heterodimerization and substrate affinity [57]. Predicted changes in secondary structure observed here could affect the D12/D1 interface, and thereby possibly alter viral gene expression. Affecting D1/D12 heterodimerization has previously been proposed as a potential therapeutic target for rational drug design [58]. We also observed an L42I substitution in the D9 subunit of the mRNA decapping enzyme (Table 1) that acts primarily on early transcripts [59]. The L42 residue appears highly conserved throughout the *Chordopoxvirinae *[59]. The D9/D10 heterodimeric decapping enzyme has been shown to decrease the levels of viral and cellular capped mRNAs and their translated products perhaps to delineate more responsive transitions between early and late stage gene expression [59].

### VP55 poly(A) polymerase

Similar to eukaryotic mRNA transcripts, viral mRNAs possess a m7G(5')pppGm cap structure and a 3' poly(A) tail. This posttranscriptional modification is carried out by the viral capping heterodimer VP39 and the heterodimeric poly(A) polymerase (PAP) protein that catalyzes 3' adenylate extension [33,34]. The large subunit of PAP is the catalytically active VP55 poly(A) polymerase and requires the small subunit (VP39) for full processivity [60]. VP39 performs dual functions and exhibits methyltransferase activity distinct from its role as a processivity factor for VP55 polyadenylation. VP55 acquires processivity by binding VP39 at a dimerization surface region distal to the VP39 methyltransferase cleft [61]. Conformational changes from this interaction occur in the VP39 methyltransferase, and VP55-VP39 interaction has been shown to positionally alter the VP55 RNA contact site [62].

**Figure 11:**
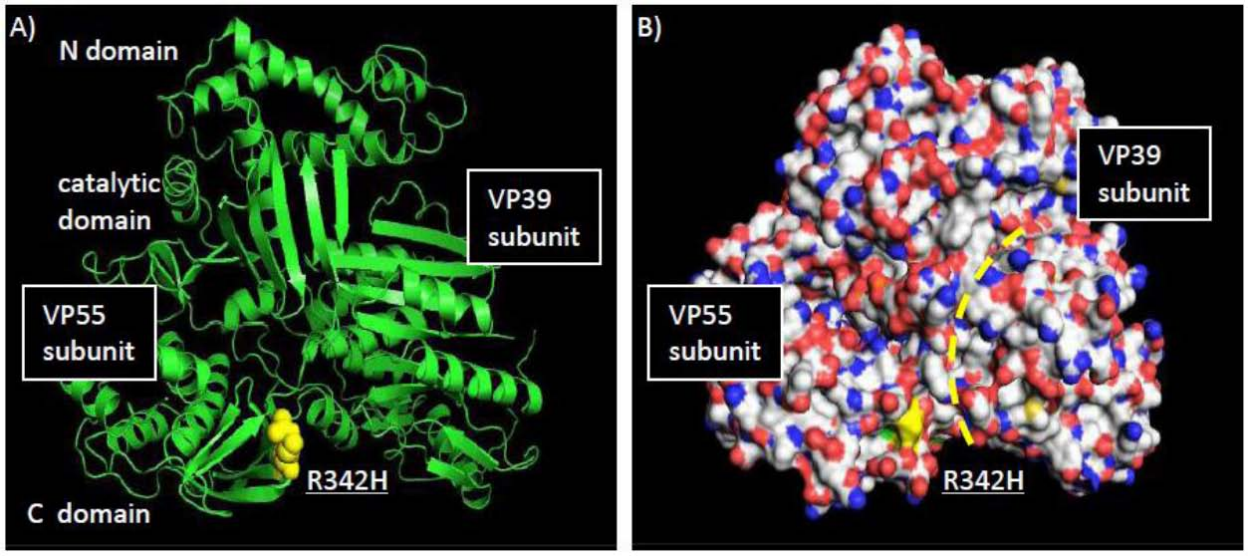
**MPV CDV-R mutations mapped onto the 3D structures of poxvirus poly-A polymerase VP55**. The R342H mutation (yellow) is presented in the ribbon diagram topology (A) and space filling model (B). Dashed yellow line (B) designates the VP55-VP39 interface.

We observed an R342H substitution (Table 1) within the VP55 C domain dimerization region interface of VP39 and VP55 (Figure 11A, B) [63]. Predictive modeling suggests that the R342H substitution decreases regional surface exposure (C domain residues 337-344) and induced a flexible coil region at the 342 locus (data not shown). Such alterations in the secondary structure within this region could alter both the VP55-VP39 interaction interface (yellow dashed line - Figure 11B) as well as the upstream proximal linker segment that supports the catalytic domain of VP55 [63]. Previously, nucleotide analogs have been postulated to negatively affect polyadenylation and early mRNA extrusion from the viral core [64]. In addition, nucleotide content within VP55 oligonucleotide primer recognition motifs may affect the timing of gene expression [64]. As a cytosine analog, CDV, if incorporated into priming sequences, could alter the primer reaction site and impart some selection pressure to maintaining effective VP55-primer recognition and subsequent processive polyadenylation of mRNA transcripts.

## Conclusion

In the current study we report the complete genomic sequence of a CDV-R strain of MPV. In addition, we present a focused and comparative bioinformatic analysis that revealed predicted alterations in topological features of functionally active domains within essential virus proteins. Previous data indicate mutations at sites 314 and 684 in the DNApol represent the primary determinants of CDV-R in VACV [15,20]. Although second-site substitutions elsewhere in the VACV genome have been implicated previously in a CDV-R clone [18], they have yet to be identified. The present study may provide clues to the location of such mutations. The MPV DNApol mutations reported here provide the first indication that CDV-R loci previously identified in VACV are perhaps conserved in fully-virulent, non vaccine strains, though such speculation must await experimental validation. Such data may inform efforts in development of Smallpox-related medical countermeasures. Any direct effects of selected mutations reported here on the resistant or attenuated phenotype of MPV must await future determination. These regions may be of particular interest for future site-directed mutagenesis studies to dissect 1) potential yet-uncharacterized mutations elsewhere in the genome that may play a role in the CDV-R phenotype, and, 2) the genetic basis of the characteristic attenuated phenotype of CDV-R poxviruses. It is possible the substitutions observed in our analysis outside the viral DNA polymerase, for example in the RNA polymerase and mRNA capping enzyme, may contribute to the resistant or attenuated phenotype of CDV-R MPV. Such changes may represent compensatory, adaptive, or attenuating variations in gene expression or replication. Also, adaptive substitutions which support a CDV-R phenotype may result in alterations in the timing of the viral gene expression program that could reduce fitness compared to wild-type yet sustain gene expression in the presence of CDV. Both adaptive and non-adaptive substitutions may also be facilitated through mutator alleles in the DNA or RNA polymerases. As DNA synthesis is a key regulator of gene expression in poxviruses, it is possible the aberrant chain extension induced by CDV may lead to diverse alterations in gene expression and replication that must be overcome by a resistant strain. The genome sequence of CDV-R MPV may inform future research into the mechanism of action of CDV as well as dissection of the phenotypic properties of resistant poxviruses. Furthermore, defining the potential contribution of substitutions in the replication complex and RNA processing machinery may inform current therapeutic development strategies and yield further insight into CDV-resistance and attenuation.

## Methods

### Viral DNA extraction, amplification and sequencing

The CDV-R strain of MPV Zaire-005 sequenced in this study was previously characterized by Smee et al 2002 (15). Poxvirus DNA were extracted from virus-infected cells utilizing the Aquapure DNA kit (Bio-Rad, Hercules, CA). Prior experiments demonstrated that the material was noninfectious after 60 min of incubation at 55°C in the Aquapure lysis buffer. The PCR amplification and sequencing primers were designed to cover the entire genome in overlapping fragments of about 500-600 bases. Primers were designed by the aid of PrimerSelect V 7.0.0 (DNASTAR, Madison, WI) using general guidelines for primers design. The criteria were as follows: Tm: 48°C to 63°C (optimum 55°C); GC content: 30-80% (optimum 50%); 3' GC clamp: none; size: 18 to 27 (optimum 20); secondary structure: 0 to 8 with a maximum of 3 bp self-complementarities at the 3' end. The melting temperature was determined according to Breslauer et al [65].

PCR was performed in 25-μl volume containing a PCR buffer (20 mM Tris-HCl, pH 8.4, 50 mM KCl), 3 mM MgCl_2_, 0.2 mM dNTP mix, 0.4 uM of each primer forward and reverse, 2 U of Platinum Taq DNA polymerase (Invitrogen Life Technologies, Carlsbad, CA), and 3 pg of DNA template. The amplification reaction was carried using the cycler PTC100 (MJ Research, Reno, NV) with the following cycling conditions: 94°C for 2 min, 45 cycles of 94°C for 30 sec, 50°C for 15 sec, and 72°C for 1 min, and one cycle of 72°C for 5 min. The PCR product was stored at 4°C until use.

Genome sequences were determined by capillary sequencing using the ABI Prism BigDye Terminator Cycle Sequencing Kit 3.1 (Applied Biosystems, Foster City, CA) and the manufacturer's instructions for PCR product sequencing. Cycle sequencing reactions were carried out on MJ Research PTC100 thermal cycler (MJ Research, Reno, NV). Labeled products were analyzed in an ABI 3700 Genetic Analyzer (Applied Biosystems). The resultant sequence reads were assembled into contigs using Lasergene 7 software, (DNASTAR). Consensus DNA sequences were obtained at least 3-fold redundancy at each base locus. The CDV-R MPV genome sequence has been deposited in GenBank under accession No. HM172544.

### Genome comparison

The MEGA 4.0 software package [66] was used for SNP/indel identification and whole genome sequence comparisons of CDV-R and WT Zaire 79-005. The genome of the seed stock used in the analysis (WT Zaire 79-005) was sequenced and compared with the genome of the final CDV-R passage.

### Protein sequence and predictive structural analysis

MEGA 4.0 was used to generate amino acid alignments. Topological feature maps containing predictive protein secondary structure analysis output was carried out using the Protean module of Lazergene (DNAstar) software under default settings. Motif surface exposure at MVP CDV-R substitution loci were estimated using the Jameson-Wolf antigenic index and the Emini method. The antigen index integrates hydropathy, conventional solvent accessibility, and flexibility to produce a linear surface contour plot [67] and provides a more comprehensive surface exposure estimate than the Emini method [68], which evaluates side-chain solvent accessibility alone (i.e., Emini plot). 3-D protein structures were modeled using PyMol software [69] and Cn3D software http://www.ncbi.nlm.nih.gov/Structure/CN3D/cn3d.shtml.

## Competing interests

The authors declare that they have no competing interests.

## Authors' contributions

JF carried out comparative genome sequence analysis, SNP identification and characterization, protein modeling and drafted the manuscript. MAI performed genome sequencing. JH developed and isolated CDV-R and WT viruses. SI conceived, directed and coordinated genome sequencing study, prepared project proposal, designed primers and performed sequence assembly. All authors read and approved the final manuscript.
